# Editorial: Protein post-translational modifications in the nervous system: from development to disease and ageing

**DOI:** 10.3389/fnmol.2024.1501719

**Published:** 2024-10-18

**Authors:** Beatriz Alvarez, Judit Symmank, Geraldine Zimmer-Bensch, Miguel Diaz-Hernandez, Patricia Franzka

**Affiliations:** ^1^Department of Biochemistry and Molecular Biology, Veterinary School, Complutense University of Madrid, Madrid, Spain; ^2^Instituto de Investigación Sanitaria del Hospital Clínico San Carlos (IdISSC), Madrid, Spain; ^3^Department of Orthodontics, University Hospital Jena, Jena, Germany; ^4^Division of Neuroepigenetics, Institute for Biology II, Rhenish-Westphalian Technical Aachen University (RWTH), Aachen, Germany; ^5^Institute of Human Genetics, Jena University Hospital, Friedrich Schiller University, Jena, Germany

**Keywords:** protein post-translational modifications (PTMs), aging, muscle, brain, development, disease, nervous system

## Abstract

PTMs are crucial for biological processes contributing to healthy organ function. Protein post-translational modifications (PTMs), such as phosphorylation (P), acetylation (Ac), SUMOylation (SUMO), S-nitrosylation (Nitro), ubiquitination (Ub) and glycosylation (Glyco), affect a wide range of cellular and biological functions as depicted in this cartoon. Perturbations lead to severe consequences for the normal function of the brain and other organs, such as muscle. Created in BioRender. Hübner (2024) BioRender.com/j49w898. 
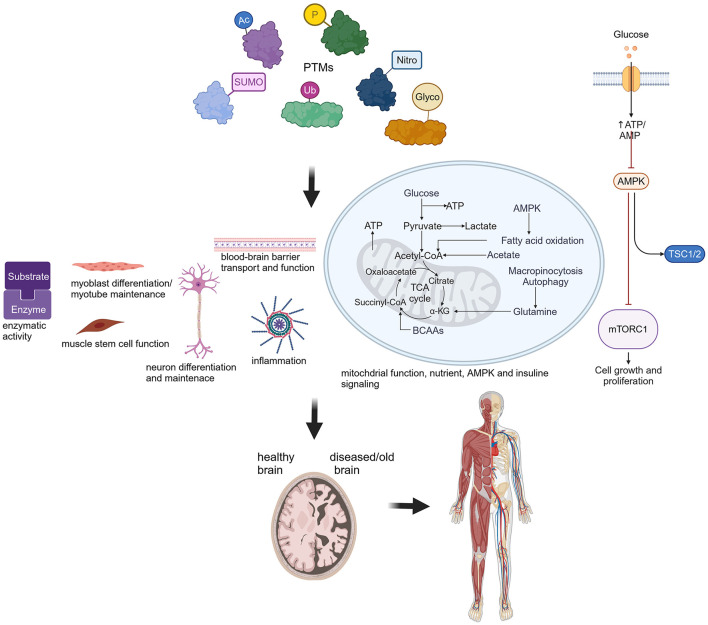

Post-translational modifications (PTMs) increase the functional diversity of the proteome by reversibly or irreversibly modifying proteins during or after their synthesis. Thereby, they contribute to the structural and functional variety of proteins, conveying a complexity to the proteome that is significantly higher than the coding capacity of the genome. Moreover, providing another level of epigenetic regulation, PTMs of histone proteins in particular contribute to the modulation of gene accessibility and specific cell expression profiles. This Frontiers Research Topic entitled “*Protein post-translational modifications in the nervous system: from development to disease and ageing*” has collected 10 contributions from experts giving new insights into our understanding of protein post-translational modifications and their involvement in disease progression, development and aging.

Three articles assessed the neurodegenerative diseases Alzheimer (AD) and Parkinson (PD) as well as key regulators of aging. Varshavskaya et al. investigated how PTMs of β-amyloid (Aβ) may affect AD. In AD, Aβ plaques accumulate within the brain and hence serve as a biomarker for this disorder. The authors provided evidence for phosphorylated Aβ_42_ as well as isomerized Aβ_42_ to cross an *in-vitro* blood-brain barrier more efficiently than unmodified Aβ_42_, and reported a different mechanism of transport. These findings could be significant for understanding AD pathogenesis and treatment, and may prove valuable in the search for biomarkers. Jonischkies et al. stressed the nuclear DBF2-related (NDR) family of serine-threonine AGC kinases as essential regulators of aging by underlining their functions, such as autophagy and inflammatory cytokine regulation, nutrient, AMPK or insulin signaling, and dysfunctions in the context of aging. Luo et al. highlighted recent findings on major PTMs, such as ubiquitination, phosphorylation, SUMOylation, acetylation, or S-nitrosylation, on mitochondrial dysfunction, a central factor in PD pathogenesis. Moreover, they discussed the potential of proteins harboring PTM sites, such as α-synuclein or VPS35, as biomarkers for PD.

In agreement with mitochondrial changes upon PTM alterations, Liu et al. showed that chronic intermittent hypoxia (CHI) alters hippocampal protein acetylation in mice. The majority of the affected proteins were involved in mitochondrial processes including oxidative phosphorylation, and the tricarboxylic acid (TCA) cycle. Mice under CHI treatment showed cognitive impairment, hippocampal lesions, glial cell activation, inhibited neurogenesis and induced inflammation.

Similarly, hippocampal lesions, plaques, and astrocytic gliosis are observed in prion disease. Bizingre et al. discussed recent findings on the impact of altered PTMs on the prion protein (PrPC), its function, and its conversion into the pathogenic variant. They also explored prion-related downstream factors as potential drug targets.

Two articles assessed the role of ubiquitination for protein function and stability in the context of neuronal development. Day et al. revealed that the Anaphase Promoting Complex (APC/C), an E3 ubiquitin ligase, regulates primary neurite formation and protein levels of the deubiquitinase ubiquitin specific peptidase 1 (USP1). Notably, SUMOylation of APC/C did not affect USP1 levels and neuron morphology. Franzka et al. assessed ubiquitination of GDP-mannose pyrophosphorylase B (GMPPB). GMPPB is important for generating GDP-mannose, which serves as a mannose donor for glycosylation. The authors showed that ubiquitination of GMPPB neither affects its stability nor its interaction with GMPPA, but modulates its enzymatic activity. Moreover, they disclosed that patient mutations could alter GMPPB ubiquitination. Thus, ubiquitination provides another level to regulate GMPPB activity and mannosylation. Notably, loss of GMPPB results in embryonic lethality as shown by Schurig et al.. Knockdown of GMPPB disrupted myoblast differentiation leading to myotube degeneration, and impaired neuron-like differentiation in N2A cells. In accordance, the authors reported that GMPPB protein abundance increased during brain and skeletal muscle development, which was accompanied by an increase in overall protein mannosylation. Another protein implicated in glycosylation disorders is the Inositol polyphosphate 5-phosphatase K (INPP5K), a phosphatase of phosphoinositides (PIs). Manzolillo et al. demonstrated that INPP5K expression increases during brain development, and its knockdown impaired neuronal-like differentiation of N2A cells, while disrupting protein glycosylation.

The final article in this Research Topic, by Majchrzak et al., explored the interactions between muscle stem cells and their immediate niche, the regulatory role of post-translational modifications (PTMs), and how these factors influence quiescence, activation, and self-renewal, particularly in the context of aging and disease.

In summary, this Research Topic highlighted the critical role of PTMs for nervous system and muscle function, as well as their interconnection during development, disease and aging.

